# Electric pulses: a flexible tool to manipulate cytosolic calcium concentrations and generate spontaneous-like calcium oscillations in mesenchymal stem cells

**DOI:** 10.1038/srep32331

**Published:** 2016-08-26

**Authors:** Marie-Amelie de Menorval, Franck M. Andre, Aude Silve, Claire Dalmay, Olivier Français, Bruno Le Pioufle, Lluis M. Mir

**Affiliations:** 1 Vectorology and Anticancer Therapies, UMR 8203, CNRS, Univ. Paris-Sud, Gustave Roussy, Université Paris-Saclay, 94805 Villejuif, France; 2 SATIE, CNRS, Ecole Normale Supérieure de Cachan, 61 av President Wilson, 94235 Cachan, France

## Abstract

Human adipose mesenchymal stem cells (haMSCs) are multipotent adult stem cells of great interest in regenerative medicine or oncology. They present spontaneous calcium oscillations related to cell cycle progression or differentiation but the correlation between these events is still unclear. Indeed, it is difficult to mimic haMSCs spontaneous calcium oscillations with chemical means. Pulsed electric fields (PEFs) can permeabilise plasma and/or organelles membranes depending on the applied pulses and therefore generate cytosolic calcium peaks by recruiting calcium from the external medium or from internal stores. We show that it is possible to mimic haMSCs spontaneous calcium oscillations (same amplitude, duration and shape) using 100 μs PEFs or 10 ns PEFs. We propose a model that explains the experimental situations reported. PEFs can therefore be a flexible tool to manipulate cytosolic calcium concentrations. This tool, that can be switched on and off instantaneously, contrary to chemicals agents, can be very useful to investigate the role of calcium oscillations in cell physiology and/or to manipulate cell fate.

Human mesenchymal stem cells (hMSCs) have the ability to differentiate into different cell types including adipocytes, chondroblasts and osteoblasts^1^,^2^,^3^. Human adipose-derived MSCs (haMSCs) are very similar to the bone marrow-derived ones^1^ but haMSCs are easier to collect making them promising candidates for cell therapy.

Even if the differentiating protocols using chemical agents to differentiate haMSC into adipocytes^4^,^5^,^6^,^7^,^8^, chondrocytes^9^,^10^,^11^ and osteocytes^12^,^13^,^14^ are well known, differentiation takes time (from 15 days to 1 month)^4^ and cannot produce all cell types. Furthermore, hMSCs spontaneously differentiate after 20 to 30 population doublings and lose their multipotency^1^,^15^,^16^.

HMSCs present spontaneous Ca^2+^ oscillations implicating (i) endoplasmic reticulum (ER) Ca^2+^ channels like the inositol 1,4,5-trisphosphate receptor (InsP_3_R) and plasma membrane (PM) Ca^2+^ channels as well as (ii) store-operated Ca^2+^ channels (SOCCs) and (iii) voltage-operated Ca^2+^ channels (VOCCs)^16^. These oscillations seem to be controlled by the Ca^2+^ release-recapture ER mechanisms amplified by the entry of external Ca^2+^ through PM Ca^2+^ channels^16^. Sun *et al*. reported that differentiated hMSCs present less Ca^2+^ oscillations than undifferentiated hMSCs and that blocking these oscillations by using a 10 V/m continuous electric field (EF) facilitates differentiation into osteogenic lineage^17^. Several other studies have pointed out the key role of the intracellular Ca^2+^ for stem cells and differentiation^18^. Moreover, various reports have shown that electromagnetic fields are able to influence the differentiation of stem cells by modulating the intracellular Ca^2+^ 19. However, the exact correlation between the intracellular calcium oscillations and the differentiation process is still unclear.

Microsecond pulsed electric field (μsPEF) of about 100 kV/m are commonly used to induce PM permeabilisation to different types of molecules (small ions^21^, drugs^22^, DNA^23^). The higher the EF amplitude, the greater the permeabilisation^24^. Since a decade, a new type of PEFs has been used: the nanosecond PEFs (nsPEFs) that are about 1 000 to 10 000 fold shorter in duration and 30 to 300 fold higher in amplitude. Application of nsPEFs can generate cytosolic Ca^2+^ peaks by permeabilising not only PM but also internal membranes such as the ER membranes, allowing the release of the Ca^2+^ stored in the ER^25^ to the cytosol.

The aim of this work was to develop a flexible way to manipulate cytosolic Ca^2+^ concentrations. This tool could be switched on and off on demand and allow to study the possibility to mimic spontaneous Ca^2+^ oscillations in haMSCs using nsPEFs or μsPEFs.

## Results

### Follow-up of the spontaneous Ca^2+^ oscillations in haMSCs

**Figure 1:**
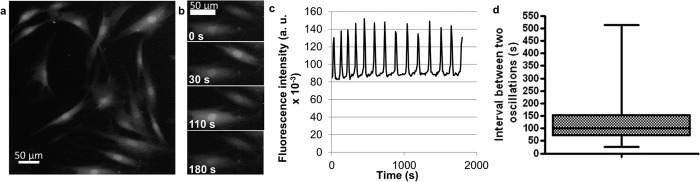
Attached haMSCs, preloaded with Fluo-4 (5 μM), presenting spontaneous Ca^2+^ oscillations in complete DMEM (with Ca^2+^). (**a**) Snapshot in epifluorescence of Fluo-4 labelled haMSCs. (**b**) Focus on two Fluo-4 labelled cells at different times of observation. The cells presented asynchronous spontaneous oscillations. (**c**) Ca^2+^ oscillations of one haMSC extracted from a movie of 30 min (one image every 10 s). The Ca^2+^ oscillations displayed regular amplitude and rhythm. (**d**) Distribution of the oscillation periods of oscillating haMSCs. 197 cells were followed from four experiments. 175 out of 197 cells (89%) displayed oscillations and for 160 of them (81%), all along the observation period. These 160 cells were used to prepare the plot. There was a large intercellular variability. The average time between two oscillations was 82 s ± 96 s, mean ± SD and the median time was 100 s. The minimum was 26 s and the maximum was 514 s.

Undifferentiated haMSCs presented asynchronous spontaneous Ca^2+^ oscillations viewable by the Fluo-4 labelling (Fig. 1a,b). Figure 1c shows the stable repetition frequency of cytosolic Ca^2+^ concentration oscillations of one cell displaying 14 peaks in about 1800 s (≈128 s between each Ca^2+^ oscillation). Even if for each cell the rhythm of the Ca^2+^ oscillations was rather stable, there was a large intercellular variability in the interval between two oscillations which is in agreement with the idea of asynchronous oscillations (Fig. 1d). The mean interval between two oscillations was 82 s ± 96 s, mean ± SD (n = 160 cells).

### Generation of Ca^2+^ peaks using nsPEFs

Since one of our goals was to mimic the spontaneous oscillations with only one single PEF of 10 ns duration and high electric field amplitude, different magnitudes of the PEFs were tested on adherent cells with or without Ca^2+^ in the external medium.

**Figure 2:**
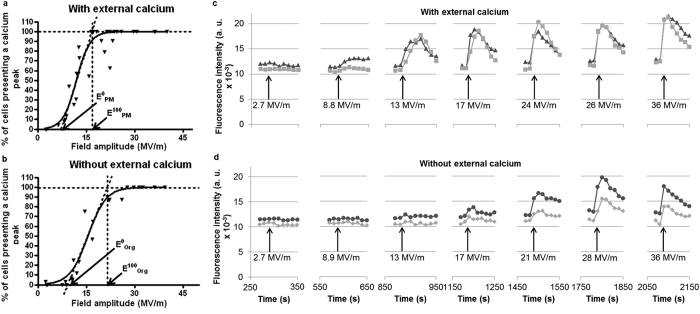
Effect of the PEF amplitude on the induced Ca^2+^ peaks with or without external Ca^2+^. (**a**,**b**) Field amplitudes needed to start to permeabilise the attached haMSCs (E^0^) and to permeabilise all the attached haMSCs (E^100^) in the presence (**a**) or in the absence of external Ca^2+^ (**b**). E^0^_PM_ and E^100^_PM_ are referring to the plasma membrane whereas E^0^_Org_ and E^100^_Org_ are referring to the organelles membranes. These experiments have been repeated at least 4 times in the presence and in the absence of Ca^2+^. 17 to 30 cells per experiment were observed for each tested electric field amplitude. Lower electric field was needed to induce Ca^2+^ peaks in the presence of external Ca^2+^ than in the absence of Ca^2+^. (**c**,**d**) Cytosolic Ca^2+^peaks recorded in two different experiments with adherent haMSCs responding to seven consecutive nsPEFs amplitudes ranging from 2.7 MV.m^−1^ to 36 MV/m^−1^. The plotted cells were representative of the general cell behaviour. (**c**) 2 cells Ca^2+^ profiles from an experiment performed in the presence of external Ca^2+^ (complete DMEM). (**d**) 2 cells Ca^2+^ profiles from an experiment performed in the absence of external Ca^2+^ (S-MEM). The shape of the beginning of the Ca^2+^ peaks was depending on the electric field amplitude.

In the presence of Ca^2+^ (Fig. 2a), a single 10 ns pulse of 7.5 MV/m was needed to induce a cytosolic Ca^2+^ increase showing a permeabilisation of the adherent haMSCs to Ca^2+^ ions. An amplitude of 16.8 MV/m was needed to induce a Ca^2+^ peak in 100% of the haMSCs. In this case, Ca^2+^ fluxes can be established across the PM and/or organelles membranes.

In the absence of external Ca^2+^ (Fig. 2b), 9.5 MV/m were needed to visualise a cytosolic Ca^2+^ increase and 21.5 MV/m were needed to observe a Ca^2+^ peak in 100% of the haMSCs. In that case, the mobilised Ca^2+^ can only be the internally stored Ca^2+^, revealing permeabilisation of organelles membranes.

In the presence of Ca^2+^, an 8.8 MV/m nsPEF induced small Ca^2+^ peaks whereas a 13 MV/m nsPEF induced a higher Ca^2+^ peak which started gradually (similar to the spontaneous oscillations). From 17 MV/m to 36 MV/m cells presented Ca^2+^ peaks starting with sharp rises (Fig. 2c).

In the absence of Ca^2+^, no effects were seen when one 10 ns nsPEF of 2.7 MV/m and 8.9 MV/m were applied (Fig. 2b,d). A nsPEF of 13 MV/m induced a small Ca^2+^ response. With nsPEFs from 17 to 21 MV/m Ca^2+^ peaks presented a slow rise at the beginning. For nsPEFs of 28 MV/m cells presented Ca^2+^ peaks with increasing amplitudes and sharp rises at the beginning. For the application of a 36 MV/m nsPEF, the amplitude of the responses slightly decreased (Fig. 2d). All together, these data show that the nsPEF amplitude needed to generate a Ca^2+^ peak was therefore lower in the presence of external Ca^2+^ showing that PM was more susceptible to electric pulses of 10 ns than organelles membranes.

**Figure 3:**
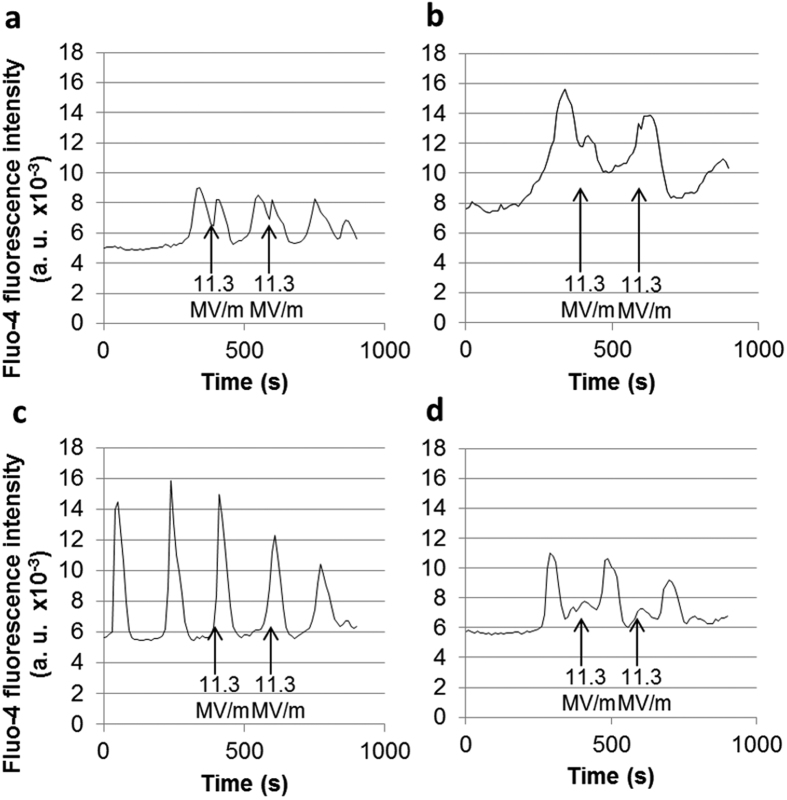
Attached haMSCs cells presenting spontaneous and induced Ca^2+^ peaks in complete DMEM. 47 cells were treated in this experiment where two nsPEFs of 10 ns were applied. 39 of them (83%) presented a Ca^2+^ peak due to the application of a nsPEF. Four of the 39 responding cells are plotted here. In all the cases, the two nsPEFs were applied at 400 s and 600 s of the recording. (**a**–**c**) Application of nsPEFs during spontaneous peaks. If the nsPEF is delivered simultaneously with the decreasing part of a spontaneous oscillation, the cell can exhibit an electrically induced Ca^2+^ peak (**a**,**b**). There was no electrically induced Ca^2+^ peak if the nsPEF was delivered during the rising part of the spontaneous oscillation (**c**). (**d**) The nsPEFs were applied between two spontaneous oscillations. The cell presented a small induced Ca^2+^ peak. Amplification (see Fig. 6) could occur (**a**) or not (**d**) at this electric field amplitude, depending on the cells. In all the cases the application of nsPEFs did not stop the spontaneous oscillations.

In Fig. 3, two nsPEFs of 10 ns and of an electric field amplitude strong enough to induce Ca^2+^ peaks (11.3 MV/m) were applied with 200 s of interval, in order to compare the electrically induced Ca^2+^ peaks to the spontaneous ones. Most of the cells responded to each nsPEF by an increase in the cytosolic Ca^2+^ concentration. With external Ca^2+^, nsPEF of 11 MV/m allowed reproducing Ca^2+^ peaks with comparable amplitude and duration as the spontaneous ones without stopping the spontaneous oscillations (Fig. 3a). If nsPEFs were delivered during the decreasing time of the spontaneous Ca^2+^ peaks, cells still showed an increase in Ca^2+^ concentration (Fig. 3a,b). But, there was no effect if the nsPEF was delivered during the rising step of the spontaneous Ca^2+^ oscillations (Fig. 3c). On Fig. 3d, each nsPEF occurred between two spontaneous Ca^2+^ oscillations and generated a Ca^2+^ peak (of smaller amplitude, in this particular cell, compared to the spontaneous oscillations) without stopping the spontaneous oscillations.

**Figure 4:**
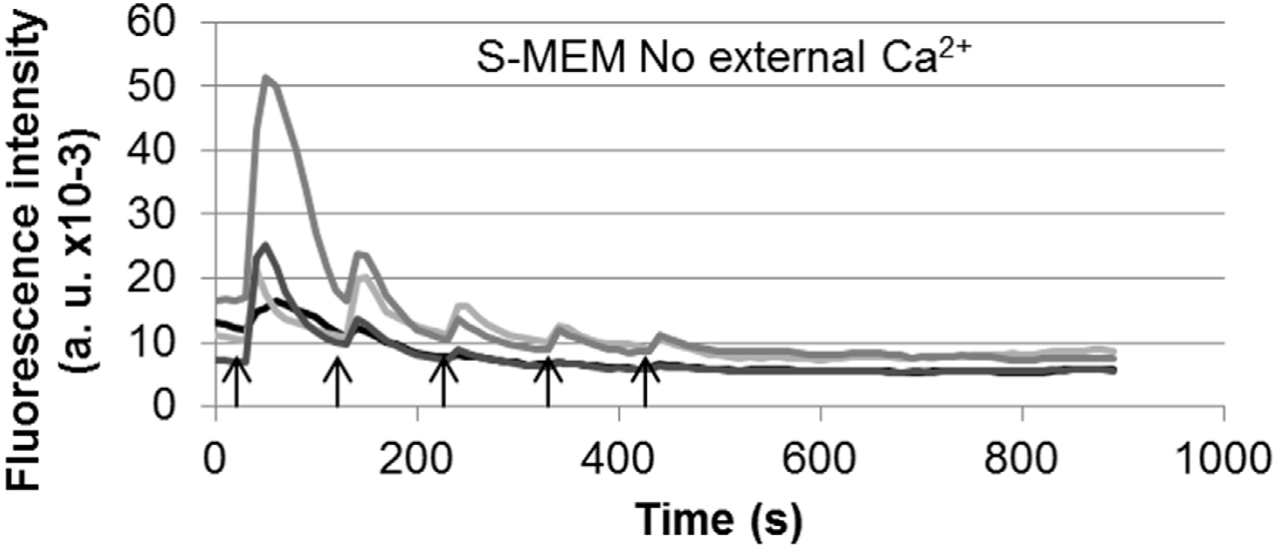
HaMSCs in suspension presenting nsPEFs induced Ca^2+^ peaks. Cells were pulsed in S-MEM. The cells are laid down on the bottom of the channel of the exposure device. The nsPEFs were delivered every 100 s. The 3 first arrows correspond to a 10 ns pulse of 20 MV/m. The last 2 arrows correspond to a 10 ns pulse of 24 MV/m. Each nsPEF induced an immediate and sharp rise in cytosolic Ca^2+^ concentration. In response to two nsPEFs of identical field strength, amplitude of the second Ca^2+^ peak was 10 fold smaller than the first one. Even a higher nsPEF amplitude (for example 24 MV/m) delivered after 3 nsPEFs of 20 MV/m did not generate a higher Ca^2+^ peak, and did not even stop the progressive decrease in Ca^2+^ peaks amplitude.

HaMSCs were also treated in suspension without external Ca^2+^ and they also presented Ca^2+^ peaks with sharp rises in response to nsPEFs due to the release of Ca^2+^ from the internal stores and mostly the ER (Fig. 4). The amplitude of the Ca^2+^ peaks induced by a train of nsPEFs decreased progressively from pulse to pulse even when nsPEFs of higher amplitudes were delivered.

### Generation of Ca^2+^ peaks using μsPEFS

**Figure 5:**
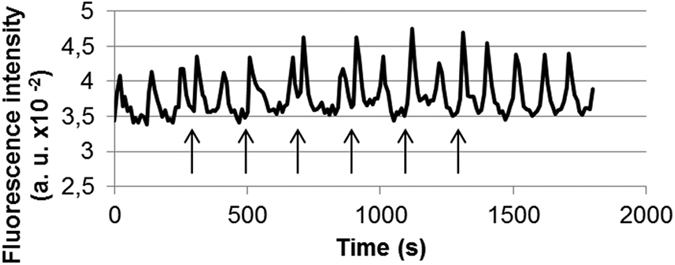
Attached haMSC presenting 100 μs PEF induced Ca^2+^ peaks (31 kV/m). The pulses were delivered every 200 s (arrows) in complete DMEM medium. There is no loss of the spontaneous oscillations and the electrically induced Ca^2+^ peaks displayed a duration and an amplitude similar to those of the spontaneous oscillations.

Reproduction of spontaneous oscillations (same amplitude and duration) was achieved using one μsPEF of 100 μs and 15–31 kV/m. However, not all the cells responded. The higher the μsPEF amplitude, the higher the percentage of cells presenting a Ca^2+^ response (up to 98% with the delivery of a μsPEF of 31 kV/m, Supplementary Table S1). Interestingly, part of the responding cells presented a slow rise at the beginning of the peak (Supplementary Table S1), like the spontaneous oscillations. The other cells exhibited a sharp rise in the cytosolic Ca^2+^ concentration (Fig. 5). The lower the μsPEF amplitude, the higher the percentage of cells presenting a slow rise at the beginning of the Ca^2+^ peaks (Supplementary Table S1). The amplitudes of the induced Ca^2+^ peaks were similar to the amplitudes of the spontaneous Ca^2+^ oscillations. The μsPEF induced Ca^2+^ peaks produced are due to external Ca^2+^ entry through the PM, since no peak was detected in the absence of external Ca^2+^ (data not shown).

Spontaneous oscillations were not interrupted by the application of six μsPEFs (Fig. 5). The addition of 30 μM of propidium iodide (PI) during the treatment or 30 min after the last electrical stimulation showed no PI uptake (data not shown) revealing that cells were still alive 30 min after the treatment and that these pulses lead to a very weak permeabilisation.

Finally, in the absence of external Ca^2+^ (S-MEM) there was no increase in Ca^2+^ fluorescence when applying one single μsPEF of 31 kV/m (data not shown) proving that external Ca^2+^ was involved in the Fig. 5 data.

## Discussion

PEFs provoke the transient permeabilization of the cells membranes. The extent of this transient electropermeabilisation (also termed electroporation) depends on the PEF amplitude, duration, number and repetition frequency. As a consequence, hydrophilic molecules, including Ca^2+^ ions, can cross the electropermeabilized membranes. We used PEFs to generate artificial Ca^2+^ peaks: we show that one nsPEFs or one μsPEFs can provoke a Ca^2+^ peak similar to the spontaneous oscillations (same amplitude and duration, depending on the electric field amplitude and on the presence or the absence of external Ca^2+^). The presence or absence of serum in the media did not change the effect of the pulse, (data not shown).

Different publications already presented Ca^2+^ release induced by nsPEFs in non-excitable cells. It was shown that 10 nsPEFs of 30 ns at 2.5 MV/m^25^ or a single 60 ns pulse of 2.5 to 10 MV/m^26^ can cause Ca^2+^ release from the ER. In the former case, nsPEF affected organelles without affecting PM Ca^2+^ channels^25^. For the first time, we report that in non-excitable cells showing spontaneous Ca^2+^ oscillations (the haMSC) a single nsPEF as short as 10 ns can cause a Ca^2+^ release and a Ca^2+^ peak that can be almost identical to one Ca^2+^ oscillation as discussed below. The shape of the haMSC spontaneous oscillations is in agreement with the hypothesis described by Kawano *et al*.^16^ (the oscillations are initiated by the release of Ca^2+^ stored in the ER and amplified by external Ca^2+^ entry through PM Ca^2+^ channels). Actually, some haMSCs were not presenting spontaneous Ca^2+^ oscillations during the observation time (11%, as indicated in the legend of Fig. 1). Most of the cells displayed Ca^2+^ oscillations of regular amplitude and rhythm, even though there was a large intercellular variability in the time between two consecutive oscillations (Fig. 1d and legend of Fig. 1). Since there is no reason for cell cycle synchronization in our cultures and since it has been shown that hMSCs display Ca^2+^ oscillations only during the G1/S phases of the cell cycle^27^, the non-oscillating cells could be the ones out of the G1 or S phases during the observation period. Interestingly, when one pulse is applied, all the exposed cells, synchronously, displayed a Ca^2+^ peak (Fig. 2a,b).

### Ca^2+^ peak generation by μsPEF

**Figure 6:**
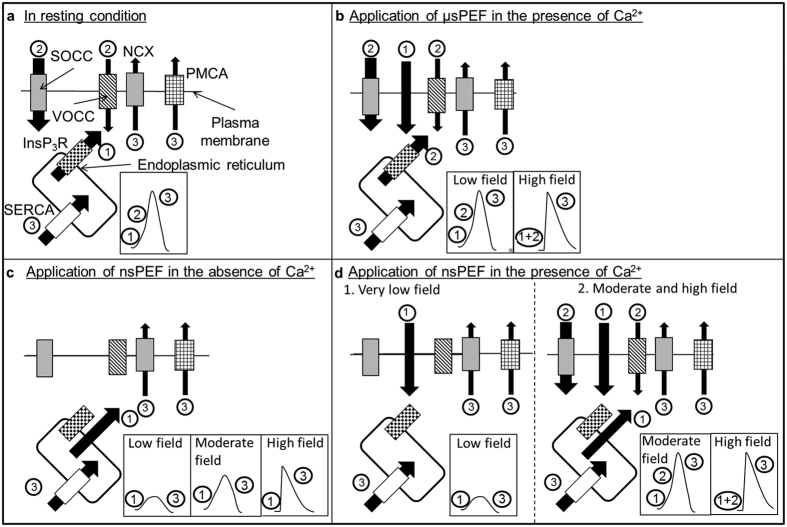
Summary scheme of the Ca^2+^ flows observed in haMSCs. (**a**) Spontaneous oscillations according to Kawano *et al*.^16^. (**b**) Ca^2+^ peak triggered by μsPEF in the presence of Ca^2+^. When a low electric field amplitude was applied (from 15 to 25 kV/m) the shape of the induced Ca^2+^ peaks was the same as the one of the spontaneous oscillations. When a higher electric field amplitude was applied, the amplitude and the duration were the same as the ones of spontaneous oscillations but the shape of the beginning of the peak was sharper than the one of spontaneous oscillations. With μsPEFs only the plasma membrane was permeabilized. (**c**) Ca^2+^ peak triggered by nsPEF in the absence of Ca^2+^. The higher the electric field strength, the higher the amplitude of the induced Ca^2+^ peak. (**d**) Ca^2+^ peak triggered by nsPEFs in the presence of Ca^2+^. At low electric field, only the plasma membrane was permeabilized. At moderate and high electric field amplitudes, the plasma membrane and the ER membrane were permeabilized. Black arrows represent Ca^2+^ flows. The number 1 corresponds to the event that initiates the calcium peak, the number 2 is the amplification of the calcium peak and the number 3 is the return to the basal state.

Present knowledge assumes that μsPEF cannot cause the permeabilisation of organelles membranes since, with μsPEF, the cell membrane shields the cell inside and the main effect of the μsPEF is therefore at the level of the plasma membrane. In the presence of external Ca^2+^, the proportion of responding cells presenting induced Ca^2+^ peaks with the same shape as the spontaneous oscillations was depending on the μsPEF amplitude (Supplementary Table S1) which can be explained by the hypothesis that, the higher the μsPEF amplitude, the more massive the Ca^2+^ entry through the μsPEF-permeabilised PM. Figure 6 summarizes the different mechanisms involved in the Ca^2+^ peaks generation. It is remarkable that each time a Ca^2+^ peak was induced by a μsPEF, it had an amplitude and a duration similar to those of the spontaneous Ca^2+^ oscillations. According to Kawano *et al*.^16^, SOCCs and VOCCS are involved in the amplification mechanism of spontaneous Ca^2+^ oscillations observed in haMSCs (Fig. 6a). It is therefore possible that a Ca^2+^-induced Ca^2+^ release (CICR) pathway involving the release of calcium from the ER and activation of VOCCS and SOCCS is also involved in the amplification of the electrically induced Ca^2+^ peaks if the initially μsPEF-induced Ca^2+^ permeabilisation is large enough (Fig. 6b). The amplification through SOCCs and VOCCs seemed to be controlled by a threshold phenomenon. But, regarding to the intercellular variability in our experiments, it was not possible to determine this threshold.

The application of low amplitude μsPEFs (15 to 25 kV/m) seems to be interesting to synchronously cause a larger proportion of cells reproducing the same shape of Ca^2+^ peaks as the spontaneous oscillations. At this electric field amplitude not all the cells responded (Fig. 6b “low field”). Application of higher amplitude μsPEFs (31 kV/m) mostly generated Ca^2+^ peaks with a sharp rise (Fig. 6b, “high field”) due to larger PM permeabilisation to Ca^2+^, without organelle permeabilisation. This description is in agreement with the classical models of cell electropermeabilisation^24^,^28^.

### Ca^2+^ peak generation by nsPEF

It is known that nsPEFs can permeabilise the ER membrane^25^ and the PM^29^,^30^, and that the electropermeabilisation occurs at the level of the lipid bilayer (according to molecular dynamics simulations^31^,^32^ and because lipid vesicles can be permeabilised)^33^,^34^.

### In the absence of external Ca^2+^

nsPEF amplitudes of more than 9.5 MV/m were needed to induce a Ca^2+^ peak and the higher the nsPEF strength, the higher the Ca^2+^ peak amplitude as well as the sharper the beginning of the peak (Fig. 2d): Ca^2+^ peaks were due to the release of internally stored Ca^2+^ (mostly in the ER) which is the only source of Ca^2+^ in this case (Fig. 6c). Considering the shape of the Ca^2+^ response, nsPEFs of about 17–21 MV/m induced Ca^2+^ responses of smaller amplitude than the spontaneous oscillations but with similar gradual increases at the beginning. For higher nsPEFs amplitudes, Ca^2+^ peaks were not only higher but moreover they started more sharply indicating again that, in this case, Ca^2+^ flowed from the internal store and no amplification occurred through SOCCs and VOCCs. Correspondingly, a depletion of the internal Ca^2+^ stores can explain the decrease in Ca^2+^ peaks amplitude in response to several nsPEFs of 20 MV/M or 24 MV/m (Fig. 4) or to the last nsPEF applied in Fig. 2d. According to Kawano *et al*.^16^,^35^, without external Ca^2+^, refilling of the calcium stores should not be possible. Furthermore, since nsPEFs also permeabilise the PM, the leakage of the cytosolic Ca^2+^ through the PM (included the Ca^2+^ released from the stores) could enhance the internal stores depletion.

### In the presence of external Ca^2+^

Ca^2+^ peaks amplitudes were either lower than or as high as the ones of the spontaneous oscillations (Fig. 6d). These data are also in agreement with the amplification mechanism suggested with the μsPEFs. The fact that some cells exposed to a nsPEF of less than 11 MV/m showed Ca^2+^ peaks of a lower amplitude than the spontaneous oscillations (Fig. 6d 1) could be due to the fact that membranes were only slightly permeabilised. Indeed, at these field amplitudes, nsPEF-caused permeabilisation should be reduced in duration or in intensity with respect to μsPEF-caused permeabilisation (under our experimental conditions, the nsPEF are 10^4^ times shorter than the μsPEF). For nsPEFs of less than 9,5 MV/m, only the external Ca^2+^ was involved. For higher nsPEF amplitudes, both the PM and the organelles membranes were permeabilised. When nsPEFs of 11 MV/m to 13 MV/m were applied, the Ca^2+^ increases were more important and the shape of the beginning of the peak was similar to that of spontaneous oscillations (Fig. 6d 2, “moderate field”). When nsPEFs of even higher amplitude were applied (from 17 to 40 MV/m) haMSCs presented Ca^2+^ peaks with sharp rising (Fig. 6d 2 “high field”). This could be explained by the fact that membranes permeabilisation is large enough to lead to a fast and massive increase in cytosolic Ca^2+^, rapidly amplified by the SOCCs and the VOCCs (Fig. 6d 2 “1 + 2”). The amplification mechanism might control the amplitude of these peaks which is similar to the amplitude of the peaks starting by a slow increase. Indeed, the peaks presenting a sharp rise in the Ca^2+^ concentration mostly reached the same maximum as the ones showing gradual increases at the beginning. Moreover, it is noteworthy that the application of nsPEF during the rising part of a spontaneous oscillation does not display any additional effect (Fig. 3c). This observation reinforces the hypothesis that, in the presence of external Ca^2+^, the spontaneous oscillations and the induced peaks are partly using the same mechanisms (involvement of SOCCs and VOCCs), explaining why there is no additive effect and why the amplitude of the induced Ca^2+^ peaks is similar to the Ca^2+^ oscillations amplitude.

A decade ago, it was thought that nsPEFs would only permeabilise organelles, but it has been shown that nsPEFs also permeabilise PM^29^. However, it is still commonly assumed that lower amplitude nsPEFs are needed to permeabilise organelles than PM because the duration of the pulse is shorter than the time necessary to charge the membrane and because the charging time of the membrane of a small organelle is shorter than the charging time of the membrane of a larger element like the entire cell. However, we show here that, with haMSCs, pulses of 10 ns duration and Ca^2+^ ions as permeabilization marker, the amplitude of the electric pulses necessary to permeabilise the PM is lower than the PEF amplitude necessary to permeabilize the organelles membrane. Interestingly, the comparison is made here using the same marker (Ca^2+^) and the same system to visualise it (Fluo-4, used under identical conditions). Our conclusions are supported by Semenov *et al*.^36^ who recently reported that.using a single longer pulse of 60 ns, it is easier to permeabilise PM than organelles in CHO cells (the permeabilisation marker was also the Ca^2+^).

The characteristics of the permeabilisation marker and the sensitivity of the techniques used to detect it are important parameters that must be taken into account. Indeed, we showed elsewhere^37^ that the notion of chemical permeabilisation strongly depends on the dye and the technique of observation used. In all the cases, haMSCs recovered in less than two minutes, meaning that Ca^2+^ could no longer be mobilised across the membranes. The data also suggest that induced PM permeabilisation or organelles membrane permeabilisation was fully reversible.

Whatever the mechanism for the rise in cytosolic Ca^2+^ concentration, the Ca^2+^ should be pumped back to the ER through SERCA pumps (sarco/ER Ca^2+^ ATPase) and/or released to the external medium through the NCX channel (Na^+^/Ca^2+^ exchanger) and PMCA (PM Ca^2+^ ATPase) as demonstrated by Kawano *et al*.^35^.

In conclusion, we developed new flexible tools to control internal Ca^2+^ concentrations on demand and to reproduce spontaneous Ca^2+^ oscillations in haMSCs using nsPEFs and μsPEFs. The important parameters (amplitude, duration and shape of the gradient in Ca^2+^ concentration) in the regulation of cell physiology by Ca^2+^ peaks are not known but with these new tools, different shapes of Ca^2+^ peaks can be produced. Interestingly, the perturbation of cell membranes is minimal and fully reversible when the electric pulses are delivered at repetition frequencies compatible with the spontaneous oscillations rates. This way to manipulate the cytosolic Ca^2+^ concentration presents the great advantage that it can be instantaneously switched on and off, which is not possible with chemical agents. Thus it is possible to manipulate cytosolic Ca^2+^ concentrations on demand by electrical manipulation and to activate or not SOCCs and VOCCs. This approach can help to understand the implication of Ca^2+^ oscillations in biological processes like cell differentiation and to determine the important parameters of the Ca^2+^ peaks in cell physiology.

## Methods

### Cells and culture conditions

HaMSCs were isolated from surgical waste of individuals undergoing elective lipoaspiration. Samples were obtained after written informed consent from all the donors, in accordance with France and European legislations^6^,^38^. The lipoaspirates were surgical waste and as such the French legislation (Art.L. 1245-2 du Code de la Santé Publique) establishes that the authorization from an ethics committee is not required. Cells were grown in DMEM (Dulbecco’s Modified Eagle Medium) supplemented with 10% foetal bovine serum, 100 U/mL penicillin and 100 μg/mL streptomycin. Cell culture chemicals were purchased from Life Technologies (Cergy Pontoise, France). Cells were propagated at 37 °C in a humidified 5% CO_2_ atmosphere by passing them every 3–4 days (one passage corresponding to one doubling time of the population). HaMSCs were isolated and characterised as reported in Liew *et al*.^39^. Briefly, HaMSCs were isolated by plastic adherence and characterised by flow cytometry analysis of specific surface antigens (positive for CD105, CD90 and CD44 and negative for CD34 and CD45, purity above 90%) and by adipogenic and osteogenic differentiation. Two different media were used in the experiments: complete DMEM (containing 1.8 mM CaCl_2_ and 10% foetal bovine serum) and S-MEM (Suspension Minimal Essential Medium, without calcium and foetal bovine serum). Serum was not used in the buffer without calcium (SMEM) since it contains some calcium.

### Electric pulses generators and exposure devices

A commercial generator purchased from FID (FID GmbH, Model FPG 10-ISM10, Burbach, Germany) was used to treat the cells. It generates trapezoidal monopolar pulses of 10 ns. The exposure devices were microchambers with parallel gold electrodes, 25 μm thick, with a gap between the electrodes of 300 μm or 150 μm. This device allows a high spatial homogeneity of the electric field (variation lower than 1.6% in z and lower than 2–10% maximum in x) as described in Dalmay *et al*.^40^.

Electric pulses of 100 μs were generated using a Cliniporator^TM^ (Igea, Carpi, Italy) connected to two parallel stainless steel rods of 1 mm diameter, 4 mm apart that were shaped to enter a 24 plate well and used as electrodes. The whole system was installed on an inverted microscope (see below).

### Imaging and images treatment

Cells were cultured on glass cover slides for at least 24 hours to obtain well spread out cells. In order to visualise the Ca^2+^ oscillations, the cells were incubated with 5 μM of the Ca^2+^ fluorescent marker, Fluo-4 AM (λex = 496 nm, λem = 515 nm) for 30 min in a humidified atmosphere with 5% CO_2_ at 37 °C in complete DMEM. To easily localise the cells, the incubation buffer also contained 375 nM of the nuclear fluorescent dye Hoechst 33342 (λex = 350 nm, λem = 461 nm). There is no interference between Fluo-4 and Hoechst 33342 fluorescences because their emission wavelengths are separated enough. The slides were washed three times with PBS (Phosphate Buffered Saline), turned upside down and placed on the top of the microchamber filled with complete DMEM or S-MEM (the cells being thus located inside the channel).

All the observations done with microsecond pulses exposure were done at 37 °C with 5% of CO_2_. Indeed, the microscope had a heating stage controlled by a Tempcontrol 37-2 digital (Pecon GmbH, Erbach, Germany) and the CO_2_ was maintained to 5% with a CTI-Controller 3700 digital (Pecon GmbH) into a Plexiglas chamber placed over the heating stage.

Images of haMSCs were taken every 10 s for 10 to 40 min with a Zeiss AxioCam Hrc controlled by the Axio Vision 4.6 software (Carl Zeiss, Oberkochen, Germany) on a Zeiss Axiovert S100 epifluorescence inverted microscope. The pulses were always delivered after at least 5 min of observation and 2 s before the next image. Bright field images and Fluo-4 and Hoechst 33342 fluorescence images were sequentially taken using shutters controlled by the AxioVision 4.6 software. The minimum opening time of the shutters for the fluorescent light was about 500 ms. In order to decrease the light energy applied on the cells, a 90% density Filter Thorlabs (NE110B, Maisons-Lafitte, France) was used.

The Hoechst 33342 was used to track the cells during videomicroscopy. Indeed, nuclei were recognised and the individual cells were tracked using the Cell Profiler software (Broad Institute, Cambridge, USA), allowing the automatic measurement of the Fluo-4 fluorescence of each cell on every image. Curves were plotted with a program in Matlab.

### Determination of the EF amplitudes needed to permeabilise Ca^2+^ internal stores and PM

Different increasing field amplitudes were applied on the same cells of a coverslide as it was difficult to compare the responses on totally different cells because of the intercellular variability. On each group of cells, 7 to 8 PEFs of 10 ns were applied with an interval of 300 s. The range of field amplitudes was from 2.5 MV/m up to 40 MV/m.

### Statistics plotting

On the box plot, minima and maxima are represented at the ends of the whiskers. The rectangle is defined by the median and by the first and the third quartile of the sample.

## Additional Information

**How to cite this article**: de Menorval, M.-A. *et al*. Electric pulses: a flexible tool to manipulate cytosolic calcium concentrations and generate spontaneous-like calcium oscillations in mesenchymal stem cells. *Sci. Rep.* **6**, 32331; doi: 10.1038/srep32331 (2016).

## Supplementary Material

Supplementary Information

## References

[b1] Lee R. H. . Characterization and expression analysis of mesenchymal stem cells from human bone marrow and adipose tissue. Cell Physiol Biochem 14, 311–324, 10.1159/000080341 (2004). 15319535

[b2] Lindroos B., Suuronen R. & Miettinen S. The potential of adipose stem cells in regenerative medicine. Stem Cell Rev 7, 269–291, 10.1007/s12015-010-9193-7 (2011). 20853072

[b3] Dominici M. . Minimal criteria for defining multipotent mesenchymal stromal cells. The International Society for Cellular Therapy position statement. Cytotherapy 8, 315–317, 10.1080/14653240600855905 (2006). 16923606

[b4] Deslex S., Negrel R., Vannier C., Etienne J. & Ailhaud G. Differentiation of human adipocyte precursors in a chemically defined serum-free medium. Int J Obes 11, 19–27 (1987). 3570635

[b5] Zuk P. A. . Multilineage cells from human adipose tissue: implications for cell-based therapies. Tissue Eng 7, 211–228, 10.1089/107632701300062859 (2001). 11304456

[b6] Zuk P. A. . Human adipose tissue is a source of multipotent stem cells. Mol Biol Cell 13, 4279–4295, 10.1091/mbc.E02-02-0105 (2002). 12475952 PMC138633

[b7] Hauner H. . Promoting effect of glucocorticoids on the differentiation of human adipocyte precursor cells cultured in a chemically defined medium. J Clin Invest 84, 1663–1670, 10.1172/JCI114345 (1989). 2681273 PMC304034

[b8] Sen A. . Adipogenic potential of human adipose derived stromal cells from multiple donors is heterogeneous. J Cell Biochem 81, 312–319, 10.1002/1097-4644(20010501)81:2<312::AID-JCB1046>3.0.CO;2-Q (2001). 11241671

[b9] Reinisch A. . Humanized system to propagate cord blood-derived multipotent mesenchymal stromal cells for clinical application. Regen Med 2, 371–382, 10.2217/17460751.2.4.371 (2007). 17635045

[b10] Hudson J. E. . A defined medium and substrate for expansion of human mesenchymal stromal cell progenitors that enriches for osteo- and chondrogenic precursors. Stem Cells Dev 20, 77–87, 10.1089/scd.2009.0497 (2011). 20446813

[b11] Bonab M. M. . Aging of mesenchymal stem cell *in vitro*. Bmc Cell Biol 7, 10.1186/1471-2121-7-14 (2006). PMC1435883 16529651

[b12] Gimble J. & Guilak F. Adipose-derived adult stem cells: isolation, characterization, and differentiation potential. Cytotherapy 5, 362–369, 10.1080/14653240310003026 (2003). 14578098

[b13] Halvorsen Y. D. . Extracellular matrix mineralization and osteoblast gene expression by human adipose tissue-derived stromal cells. Tissue Eng 7, 729–741, 10.1089/107632701753337681 (2001). 11749730

[b14] Halvorsen Y. C., Wilkison W. O. & Gimble J. M. Adipose-derived stromal cells–their utility and potential in bone formation. Int J Obes Relat Metab Disord 24, Suppl 4, S41–S44 (2000). 11126240 10.1038/sj.ijo.0801503

[b15] Wall M. E., Bernacki S. H. & Loboa E. G. Effects of serial passaging on the adipogenic and osteogenic differentiation potential of adipose-derived human mesenchymal stem cells. Tissue Eng 13, 1291–1298, 10.1089/ten.2006.0275 (2007). 17518709

[b16] Kawano S. . Characterization of Ca(2+) signaling pathways in human mesenchymal stem cells. Cell Calcium 32, 165–174, S0143416002001240 (2002). 12379176 10.1016/s0143416002001240

[b17] Sun S., Liu Y., Lipsky S. & Cho M. Physical manipulation of calcium oscillations facilitates osteodifferentiation of human mesenchymal stem cells. FASEB J 21, 1472–1480, 10.1096/fj.06-7153com (2007). 17264165

[b18] Tonelli F. M. . Stem cells and calcium signaling. Advances in experimental medicine and biology 740, 891–916, 10.1007/978-94-007-2888-2_40 (2012). 22453975 PMC3979962

[b19] Petecchia L. . Electro-magnetic field promotes osteogenic differentiation of BM-hMSCs through a selective action on Ca(2+)-related mechanisms. Scientific reports 5, 13856, 10.1038/srep13856 (2015). 26364969 PMC4568470

[b20] Golzio M. . [Calcium and electropermeabilized cells]. Journal de la Societe de biologie 197, 301–310 (2003). 14708352

[b21] Frandsen S. K. . Direct therapeutic applications of calcium electroporation to effectively induce tumor necrosis. Cancer Res 72, 1336–1341, 10.1158/0008-5472.CAN-11-3782 (2012). 22282658

[b22] Poddevin B., Orlowski S., Belehradek J. Jr. & Mir L. M. Very high cytotoxicity of bleomycin introduced into the cytosol of cells in culture. Biochemical pharmacology 42 Suppl, S67–S75 (1991). 1722669 10.1016/0006-2952(91)90394-k

[b23] Andre F. M. & Mir L. M. Nucleic acids electrotransfer *in vivo*: mechanisms and practical aspects. Current gene therapy 10, 267–280 (2010). 20557285 10.2174/156652310791823380

[b24] Schwan H. P. Electrical properties of tissue and cell suspensions. Advances in biological and medical physics 5, 147–209 (1957). 13520431 10.1016/b978-1-4832-3111-2.50008-0

[b25] Vernier P. T. . Calcium bursts induced by nanosecond electric pulses. Biochem Bioph Res Co 310, 286–295, 10.1016/j.bbrc.2003.08.140 (2003). 14521908

[b26] Joshi R. P. . Simulations of intracellular calcium release dynamics in response to a high-intensity, ultrashort electric pulse. Phys Rev E Stat Nonlin Soft Matter Phys 75, 041920 (2007). 17500934 10.1103/PhysRevE.75.041920

[b27] Resende R. R. . Influence of spontaneous calcium events on cell-cycle progression in embryonal carcinoma and adult stem cells. Biochim Biophys Acta 1803, 246–260, 10.1016/j.bbamcr.2009.11.008 (2010). 19958796

[b28] Kotnik T., Pucihar G. & Miklavcic D. Induced Transmembrane Voltage and Its Correlation with Electroporation-Mediated Molecular Transport. J Membrane Biol 236, 3–13, 10.1007/s00232-010-9279-9 (2010). 20617432

[b29] Silve A., Leray I. & Mir L. M. Demonstration of cell membrane permeabilization to medium-sized molecules caused by a single 10 ns electric pulse. Bioelectrochemistry, 10.1016/j.bioelechem.2011.10.002 (2011). 22074790

[b30] Vernier P. T., Sun Y. & Gundersen M. A. Nanoelectropulse-driven membrane perturbation and small molecule permeabilization. Bmc Cell Biol 7, 37, 10.1186/1471-2121-7-37 (2006). 17052354 PMC1624827

[b31] Tieleman D. P. The molecular basis of electroporation. BMC biochemistry 5, 10, 10.1186/1471-2091-5-10 (2004). 15260890 PMC489962

[b32] Vernier P. T. & Ziegler M. J. Nanosecond field alignment of head group and water dipoles in electroporating phospholipid bilayers. The journal of physical chemistry. B 111, 12993–12996, 10.1021/jp077148q (2007). 17949035

[b33] Breton M., Delemotte L., Silve A., Mir L. M. & Tarek M. Transport of siRNA through lipid membranes driven by nanosecond electric pulses: an experimental and computational study. Journal of the American Chemical Society 134, 13938–13941, 10.1021/ja3052365 (2012). 22880891

[b34] Mauroy C. . Giant lipid vesicles under electric field pulses assessed by non invasive imaging. Bioelectrochemistry 87, 253–259, 10.1016/j.bioelechem.2012.03.008 (2012). 22560131

[b35] Kawano S., Otsu K., Shoji S., Yamagata K. & Hiraoka M. Ca(2+) oscillations regulated by Na(+)-Ca(2+) exchanger and plasma membrane Ca(2+) pump induce fluctuations of membrane currents and potentials in human mesenchymal stem cells. Cell Calcium 34, 145–156 (2003). 12810056 10.1016/s0143-4160(03)00069-1

[b36] Semenov I., Xiao S. & Pakhomov A. G. Primary pathways of intracellular Ca(2+) mobilization by nanosecond pulsed electric field. Biochim Biophys Acta 1828, 981–989, 10.1016/j.bbamem.2012.11.032 (2013). 23220180 PMC3560326

[b37] de Menorval M. A., Mir L. M., Fernandez M. L. & Reigada R. Effects of dimethyl sulfoxide in cholesterol-containing lipid membranes: a comparative study of experiments in silico and with cells. PloS one 7, e41733, 10.1371/journal.pone.0041733 (2012). 22848583 PMC3404987

[b38] Guilak F. . Clonal analysis of the differentiation potential of human adipose-derived adult stem cells. J Cell Physiol 206, 229–237, 10.1002/Jcp.20463 (2006). 16021633

[b39] Liew A. . Robust, efficient and practical electrogene transfer method for human Mesenchymal Stem Cells using square electric pulses. Human gene therapy methods, 10.1089/hgtb.2012.159 (2013). PMC3798228 23931158

[b40] Dalmay C., De Menorval M. A., Francais O., Mir L. M. & Le Pioufle B. A microfluidic device with removable packaging for the real time visualisation of intracellular effects of nanosecond electrical pulses on adherent cells. Lab on a chip 12, 4709–4715, 10.1039/c2lc40857k (2012). 23037002

