# Supplementary material for: Electric pulses: a flexible tool to manipulate cytosolic calcium concentrations and generate spontaneous-like calcium oscillations in mesenchymal stem cells
Source: Sci Rep. 2016 Aug 26;6:32331. doi: 10.1038/srep32331 (PMC4999807; doi:10.1038/srep32331)
Supplement: Supplementary Information [file srep32331-s1.pdf]

<u>Electric pulses: a flexible tool to manipulate cytosolic calcium concentrations and generate spontaneous-like calcium oscillations in mesenchymal stem cells</u>

Marie-Amelie de Menorval, Franck M. Andre, Aude Silve, Claire Dalmay, Olivier Français, Bruno Le Pioufle and Lluis M. Mir.

Supplementary Information

Electric field	Number of exposed cells	% of responding cells	% of the responding cells showing a gradual increase in the Ca^{2+} concentration
15 kV/m	216	42%	33%
20 kV/m	216	84%	25%
25 kV/m	142	96%	22.5%
31 kV/m	199	98%	13%

<u>Table S1:</u> Percentages of cells presenting Ca^{2+} peaks and percentages of responding cells showing a gradual increase in the Ca^{2+} concentration at the beginning of the peak in response to low electric fields µsPEF. The higher the electric field, the higher the percentage of cells presenting an induced Ca^{2+} peak and the lower the percentage of responding cell showing a gradual increase at the beginning of the peak. For each µsPEF amplitude 2 independent experiments were performed.
